# Level of evidence in wrist ligament repair and reconstruction research: a systematic review

**DOI:** 10.1186/s40634-018-0135-7

**Published:** 2018-06-07

**Authors:** Jonny K. Andersson, Bo Rööser, Jón Karlsson

**Affiliations:** 1 Department of Hand Surgery, SportsMed, Carlanderska Hospital, SE-405 45 Göteborg, Sweden; 2 Department of Orthopaedics, Institute of Clinical Sciences, The Sahlgrenska Academy, University of Gothenburg, Göteborg, Sweden; 3 Varberg, Sweden; 4 Department of Orthopaedics, Sahlgrenska University Hospital, Mölndal, Sweden

**Keywords:** Scapholunate ligament, Lunotriquetral ligament, Triangular Fibrocartilage complex, Repair, Level of evidence, Impact factor

## Abstract

There have been numerous studies on surgery of wrist ligament injuries, but a quick assessment reveals few with a high level of evidence (LoE). The primary aim of this study was to categorize the study type and LoE of studies on repair and reconstruction of the scapholunate ligament, the lunotriquetral ligament and the triangular fibrocartilage complex by applying the LoE rating system proposed by the Oxford Centre for Evidence-Based Medicine. The secondary aims were to evaluate the journal- and geographic- distribution of the included studies.

An electronic literature search of articles published 1985–2016, in PubMed, Embase, and Cochrane Library was carried out in May 2016 and updated in April 2017. Therapeutic studies written in English were included. The PRISMA checklist guided the extraction and reporting of data.

A total of 1889 studies were analyzed, of which 362 were included. Three journals represented 40% of the included studies and American authors dominated.

Most studies (97%) had low LoE (IV-V). No studies of LoE I-II were found. There is insufficient evidence to recommend one technique over the other in terms of wrist ligament surgery in clinical practice. There is an immense lack of comparison studies with high level of evidence in the area of wrist ligament repair and reconstruction.

**Electronic supplementary material:**

The online version of this article (10.1186/s40634-018-0135-7) contains supplementary material, which is available to authorized users.

## Introduction

Wrist ligament injuries are known to result in persistent pain, instability, reduced grip force and range of motion, as well as degenerative osteoarthritis. Ligament repair and reconstruction has become a highly technical developing area in wrist surgery in the last years. There are still controversies in the recommendations on surgical preferences in terms of injuries to the scapholunate ligament (SLL), the lunotriquetral ligament (LTL), the triangular fibrocartilage complex (TFCC) and instability of the distal radioulnar joint (DRUJ).

In 1986, Sackett (Sackett, 1986) proposed a system for grading different levels of medical evidence and introduced the concept of evidence-based medicine (EBM). As a concept and practice, EBM has been implemented in most if not all fields of medicine today, and wrist surgery is no exception. The level of evidence grading system can be found in the Oxford Centre for Evidence-Based Medicine (CEBM) website (http://www.cebm.net). The system categorizes a study from I to V on the basis of its design and as 1 of 4 different types on the basis of its content (therapeutic, prognostic, diagnostic and economic) (Table 1). The scale is built according to a hierarchy in which level I is the highest level of evidence, which includes high quality, randomized controlled trials (RCTs); and level V, which is the lowest level of evidence, so-called expert opinion. The grading system is widely accepted and used by most orthopaedic journals, as it ensures that the best available evidence is used in patient care and it is a fundament of EBM.

**Table 1:** Levels of evidence for primary research question on *therapeutic* studies

Level I	High-quality RCT with statistically significant difference or no statistically significant difference but narrow confidence intervals.	Systematic review of level I RCTs (and study results were homogenous)
Level II	Lesser-quality RCT (e.g. 80% follow up, no blinding, or improper randomization). Prospective comparative study.	Systematic review of level II studies or level I studies with inconsistent results.
Level III	Case-control study. Retrospective comparative study.	
Level IV	Case series.	
Level V	Expert opinion. Case report. Technical report.	

Considering the high number of studies, a quick assessment of the studies on wrist ligament repair and reconstruction reveals very few with a high level of evidence. Still, recommendations and guidelines in surgical treatment of SLL (Garcia-Elias et al., 2006; Kitay & Wolfe, 2012), LTL (van de Grift & Ritt, 2016) and TFCC/DRUJ injuries (Anderson et al., 2008; Zimmerman & Jupiter, 2014) are widely spread, as well as expert opinions. However, some questions are indicated. The most important of those are; *How strong are in fact our treatment recommendations for patients with wrist ligament injuries?* Up to this date, the patient’s needs and the surgeon’s specific skills seem to have had a great impact on choosing type of surgical treatment for SLL, LTL and TFCC/DRUJ injuries.

The recommendations in terms of diagnostics of wrist ligament injury are clear. Wrist arthroscopy is the preferred diagnostic technique with sufficient conclusive properties when it comes to wrist ligament injuries (Andersson et al., 2015; Hobby et al., 2001). In terms of surgical treatment of SLL, LTL and TFCC/DRUJ injuries, there are some strong recommendations but still many controversies – therefore this study attempts to analyse on what basis the therapeutic recommendations rests.

The primary aim of this study was to categorize the study type and level of evidence of studies on repair (suture and reinsertion) and reconstruction of SLL, LTL and TFCC/DRUJ injuries by applying the level of evidence rating system proposed by the Oxford Centre for Evidence-Based Medicine. The secondary aims were to evaluate the journal distribution and geographic distribution of the included studies and to evaluate the level of evidence over time.

## Methods

### Protocols

This systematic review was conducted according to the Preferred Reporting Items for Systematic Reviews and Meta-Analyses (PRISMA) statement (Moher et al., 2009) and follows The Cochrane Handbook for systematic Reviews of Interventions.

### Eligibility criteria

Therapeutic studies that report on wrist ligament (SLL, LTL and TFCC/DRUJ) and instability repair (suture and re-insertion) and reconstruction with clinical outcome measurements related to the ligament repair or reconstruction were included. Articles with mixed cohorts with some patients operated on with ligament repair and others operated on with other methods were included. Expert opinions, case reports and technical reports on surgery for wrist ligament injuries and instability were also included. Studies on anatomical reconstruction of the TFCC and DRUJ (Adams & Berger, 2002), other ligament reconstructions and debridement plus pinning/shrinkage were included.

Studies on animals, cadavers, diagnostic tools, economics, epidemiology, imaging, diagnostic or anatomic studies without clinical outcome; rehabilitation protocols and studies on skeletally immature populations were excluded. Studies on perilunate dislocations, midcarpal instability, ulnar shortening osteotomy, bone corrective surgery, capsulodesis without concomitant ligament repair, pinning only, arthroscopic or open debridement alone without repair or shrinkage, interosseous membrane (IOM) reconstruction, salvage procedures (4-Cornerfusion, Proximal Row Carpectomy etc), partial fusions and implant arthroplasty were excluded. The purpose of the study was to analyse the level of evidence of studies on repair (suture and reinsertion) and reconstruction exclusively. Studies not written in English, comments, authors´ reply, letters to the editor, book chapters and instructional courses were also excluded.

No outcome measures were extracted from the studies, as this was not the aim of this systematic review.

### Information sources and search

An electronic literature search of articles published between January 1, 1985 and May 24, 2016, in PubMed, Embase, and Cochrane Library was carried out in the end of May 2016, by an expert in electronic search strategies, at the Sahlgrenska University Hospital Library. Therapeutic studies written in English that report on wrist ligament repair and reconstruction with clinical outcome measurements related to the ligament repair or reconstruction were included, as well as LoE V publications on wrist ligament repair (suture and reinsertion) and reconstruction. The Preferred Reporting Items for Systematic Reviews and Meta-Analyses (PRISMA) (Moher et al., 2009) checklist guided the extraction and reporting of data. Categorization and implementation of the level of evidence and journal distribution were performed. The systematic electronic search was updated on April 28, 2017, in order to identify newly published studies that were eligible for inclusion.

No manual search of recently published articles in pertinent journals was undertaken. Corresponding authors were not contacted for additional information. The complete electronic search strategies (initial and updated) are described in the Additional file 1.

The impact factors of the journals were acquired from the Thomson Reuters Journal Citation Reports (JCR) database (ISI Web of Knowledge, http://www.webofknowledge.com). The time span in which the impact factors were able to be acquired was from 2008 to 2015/16. The Eigenfactor (Bergstrom et al., 2008) of the journals were acquired from http://www.eigenfactor.org. The time span in which the Eigenfactors were able to be acquired was from 1997 to 2013.

### Study selection

The first (JKA) and second (BR) author performed the study selection. All articles, generated by the electronic search, were screened by reading the title and abstract. The articles were validated in duplicate. The first author double-checked the extracted data by processing the included studies once again, with full agreement with the prior data selection and extraction.

If initial screening failed to provide sufficient information for the purpose of inclusion or exclusion, the full text of the article was always assessed by paper copies. In terms of 35 studies, it was necessary to retrieve full text to be able to decide about inclusion or exclusion. The investigator was not blinded to the names of authors or journals during the screening and data-extraction process.

### Data extraction and data items

#### Data extraction

Data extraction was performed according to the PRISMA (Moher et al., 2009) checklist by obtaining data on a standardized extraction sheet. Data extraction sheets with pre-determined questions were used.

Disagreements on study selection, data extraction, and assessment were resolved by discussion with the third author (JK).

#### Data items

Data items obtained from the included articles were as follows: participants, interventions, comparisons, outcomes, study design and setting (PICOS), publication year, authors, sample size, follow up time, journal, the journal impact factor, Eigenfactor and the level of evidence (LoE). In terms of eight papers, it was necessary to retrieve full text to be able to decide the level of evidence, according to the level of evidence rating system proposed by the Oxford Centre for Evidence-Based Medicine.

The level of evidence attributed to the study by the publishing journal was taken into account when assessing the study. However, when there was disagreement between the level of evidence as assessed by the publishing journal and the level of evidence as assessed by the researchers, the assessment by the researchers was used. Standardized, studies with ≤ four patients, were classified as case reports (LoE V) and studies with ≥8 patients, were classified as a case series (LoE IV).

Eigenfactor (Bergstrom et al., 2008) score is correlated with the impact factor and the total citation count for medical journals, but these metrics provide substantially different information. For a given number of citations, citations from more significant journals will result in a higher Eigenfactor score. To have the ability to analyse the importance of journals choosen for publications of studies in terms of surgery for wrist ligament injuries, we applied the Eigenfactor to the included studies. Originally Eigenfactor scores were measures of a journal’s importance; it has been extended to author-level. It can also be used in combination with the so-called h-index (Hirsch, 2005) to evaluate the work of individual scientists. However, we found that frequency tables for geographic distribution, combined with the level of evidence, was more illustrative in the present study.

## Results

### Study characteristics

**Fig. 1:**
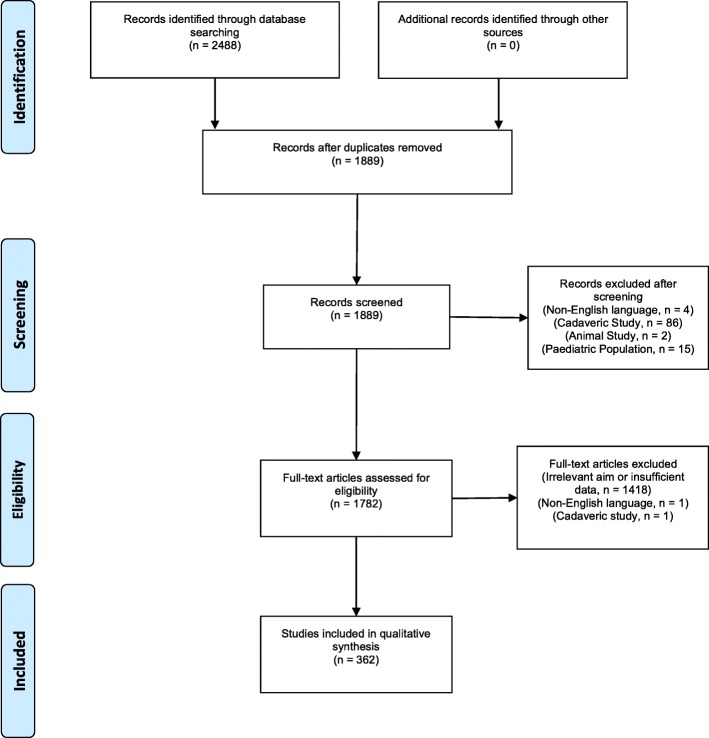
Flow diagram of inclusion and exclusion (initial search May 24, 2016)

A total of 1889 studies were analyzed, of which 362 were included. Figure 1 shows the flowchart of inclusion and exclusion of this systematic review.

### Outcome measures

#### Level of evidence

**Fig. 2:**
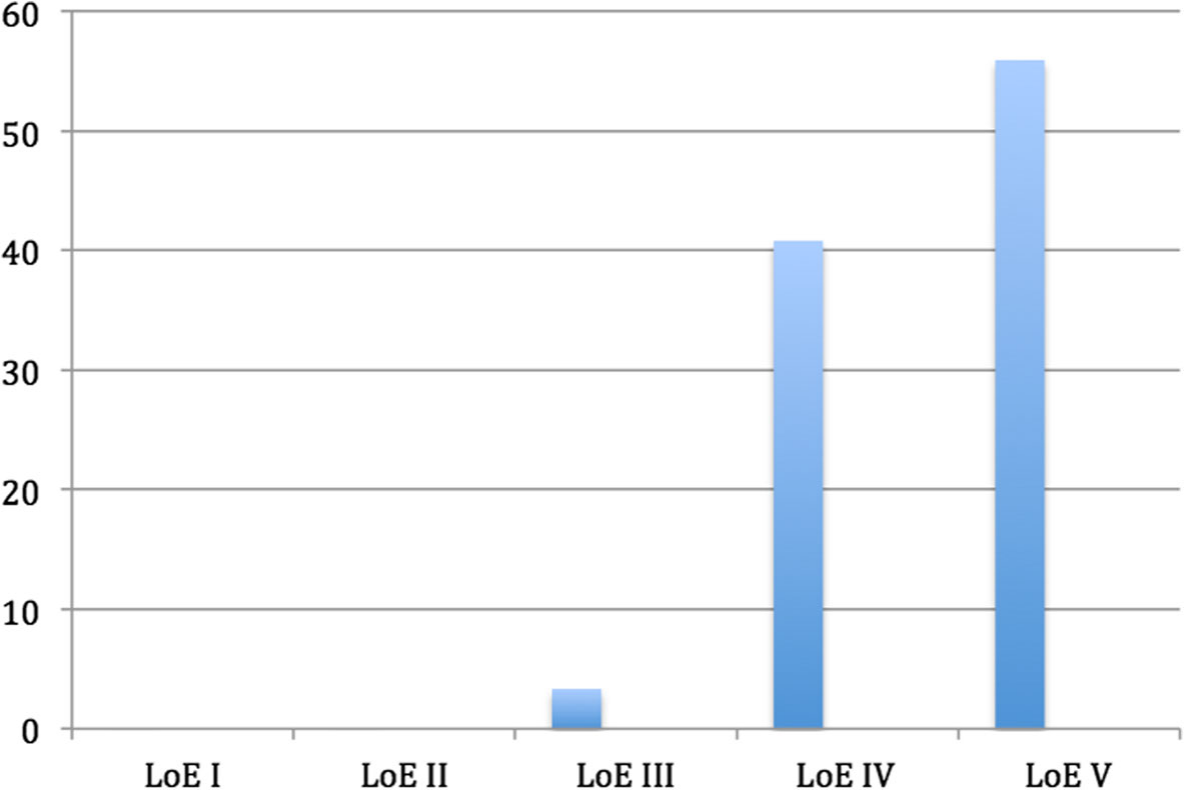
Distribution (%) of the level of evidence of the included studies

Analysis of the study types revealed that expert opinions, LoE V (*n* = 203) was the most frequent study type. Randomized controlled trials (level of evidence I) and level of evidence (LoE) II studies represented 0% (*n* = 0) of the sample. Thus, not one LoE I or II study could be found during 30 years of research in the area of wrist ligament injury surgery. LoE III studies represented 3.0% (*n* = 11), LoE IV studies represented 40.9% (*n* = 148), and LoE V 55.1% (n = 203). The distribution (%) of the included studies in terms of level of evidence is displayed in Fig. 2. Disagreement between the level of evidence as assessed by the publishing journal and the level of evidence as assessed by the researchers was noticed in two cases.

In general the included studies included small sample sizes (in average 23 patients) and rather short follow up (in average 37 months) – only 2% displayed long-term follow up (> 10 years) and 7% mid-term follow up (6–8 years). 91% of the included studies displayed short-term follow up (2 years). Over 80% of the LoE IV studies were retrospective.

### Distribution of publications

**Fig. 3:**
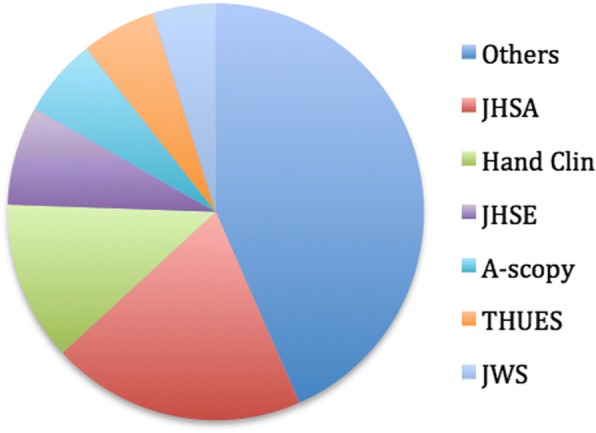
Distribution of publications in journals (percentage)

Six journals; *J Hand Surg Am*, *J Hand Surg Br/Eur*, *Hand Clin*, *Arthroscopy, Tech Hand Up Extrem Surg and J Wrist Surg* represented 56.4% (*n* = 204) of the included studies. The distribution of publications in different journals (numbers, percentage) and their impact factors are displayed in Fig. 3 and Table 2. In fact, three journals; *J Hand Surg Am, J Hand Surg Br/Eur* and *Hand Clin* represented 40% of the included studies (*n* = 145).

**Table 2:** Distribution of publications in journals (numbers, percentage) and their impact factors

Journal	Impact factor (2015)	Number (Total *n* = 362)	%
J Hand Surg Am (JHSA)	1.64	72	19.9
Hand Clin	0.70	46	12.7
J Hand Surg Eur/Br (JHSE)	1.87	28	7.7
Arthroscopy (A-scopy)	3.72	21	5.8
Tech Hand Up Extrem Surg (THUES)	1.36 (2010)	20	5.5
J Wrist Surg (JWS)	No data	17	4.7
Subtotal		204	56.4
Others		158	43.6

### Trends

Only 21.5% (*n* = 78) of the studies (66 authors) were published before 2000, in which 0% represented LoE I and II studies, 1.4% (n = 1) represented LoE III studies, 31.9% (*n* = 23) represented LoE IV studies and 66.7% (*n* = 48) represented LoE V. Almost four fifthts of the included studies were published between 2000 and 2016. There was no trend toward higher level of evidence over time. The updated electronic search performed on April 28, 2017 did not generate any new studies with high level of evidence (LoE I-II) eligible for inclusion.

**Fig. 4:**
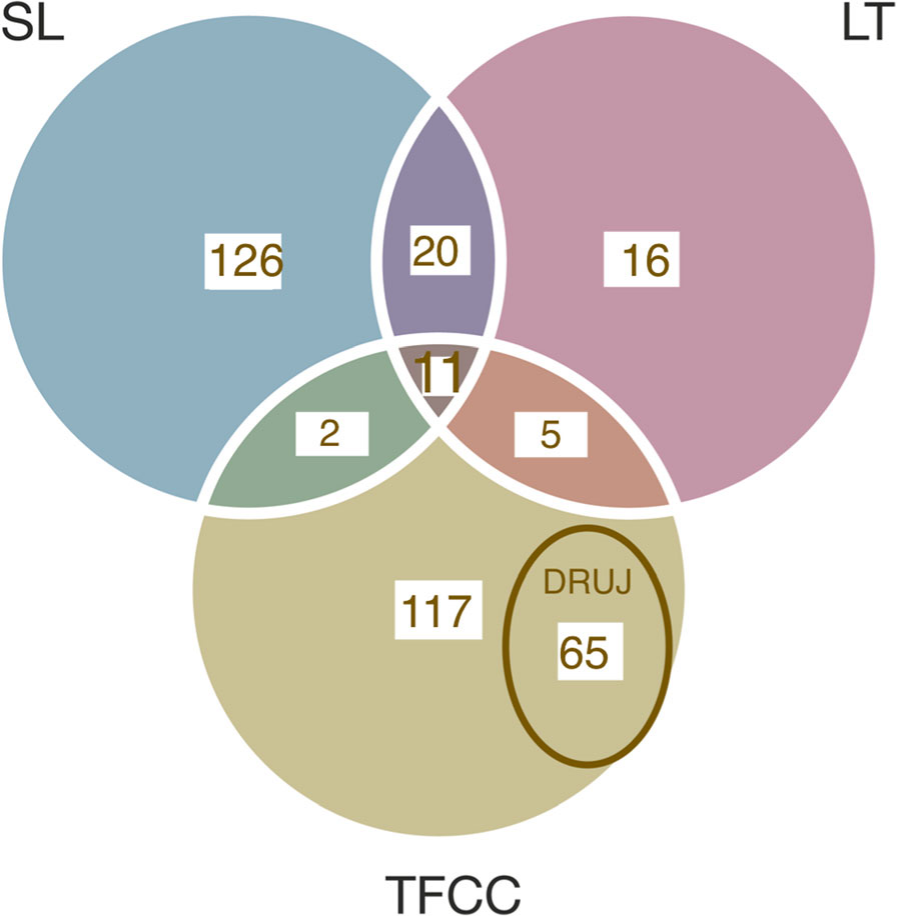
The distribution (n) of the included studies on different wrist ligaments injuries and injury combinations. (SL = scapholunate ligament, LT = Lunotriquetral ligament, TFCC = Triangular fibrocartilage complex, DRUJ = Distal radioulnar joint)

Studies on TFCC (*n* = 117) and TFCC/DRUJ (*n* = 65) as well as SLL (*n* = 126) dominate in the literature. The distribution of studies on different wrist ligaments injuries and injury combinations are displayed in the Venn diagram below (Fig. 4).

**Fig. 5:**
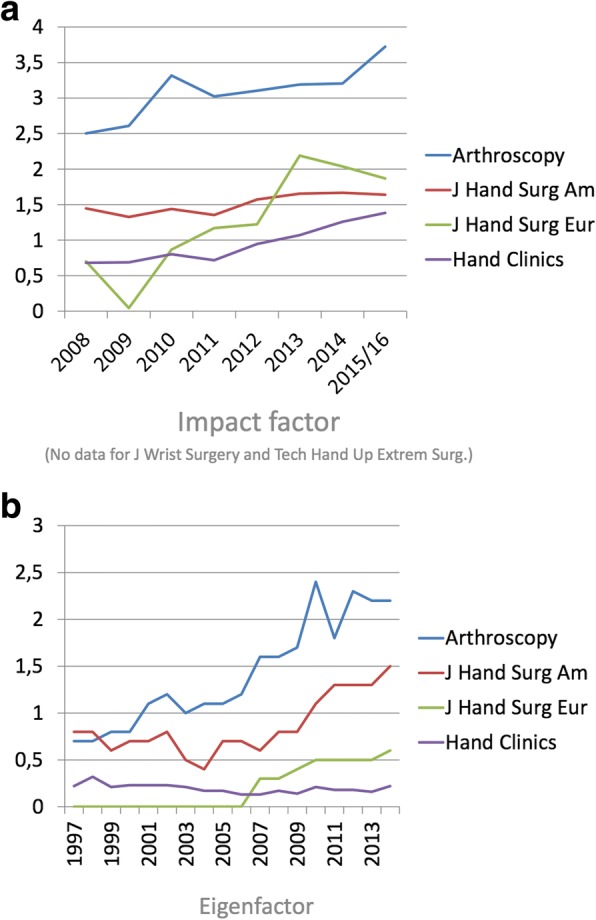
**a** and **b**. The Impact factor and Eigenfactor for the most common journals. No data were available for *Tech Hand Up Extrem Surg and J Wrist Surg*

The Impact factor and Eigenfactor for the most common journals are shown in Fig. 5a and b. No data were available for *Tech Hand Up Extrem Surg and J Wrist Surg*.

In general the Impact factor was relatively low, slightly increasing over years. The Eigenfactor followed the Impact factor.

### Geographic distribution

Frequency tables for geographic distribution revealed that the United States was the country with the highest number of publications. Authors from the United States represented 53.8% of all included publications. Authors from the United States published 130 (63.7%) of the 204 included studies in the six most common journals (*J Hand Surg Am*, *J Hand Surg Br/Eur*, *Hand Clin*, *Arthroscopy, Tech Hand Up Extrem Surg and J Wrist Surg*). Frequency tables for geographic distribution combined with the level of evidence for the six most common journals are displayed in Table 3.

**Table 3:** Frequency table for geographic distribution combined with the level of evidence for the six most common journals

Table 3 Journal	Country							LoE III	LoE IV	LoE V
	*United States* *(n - %)*	*Japan* *(n)*	*Italy/ Spain/ France* *(n)*	*United Kingdom* *(n)*	*Switzerland/ Germany/ Belgium/ Netherlands (n)*	*Other Europe* *(n)*	*Other Asia etc (n)*			
								(n-%)	(n-%)	(n-%)
*J Hand Surg Am (n = 72)*	47	6	4	1	7	2	5	5	33	34
	65%							7%	46%	47%
*Hand Clin* *(n = 46)*	40	1	2	0	1	1	1	0	5	41
	87%								11%	89%
*J Hand Surg Br/Eur* *(n = 28)*	8	3	8	2	4	1	2	1	18	9
	29%							4%	64%	32%
*Arthro-scopy* *(n = 21)*	10	1	2	0	2	1	5	0	11	10
	48%								52%	48%
*Tech Hand Up Extrem Surg (n = 20)*	11	3	1	0	1	0	4	0	5	15
	55%								25%	75%
*J Wrist Surg (n = 17)*	4	3	4	0	3	1	2	0	14	3
	24%								82%	18%

## Discussion

To our knowledge, this is the first systematic review on categorizing the study type and level of evidence of studies on repair and reconstruction of wrist ligament injuries.

### Level of evidence

This systematic review shows that most therapeutic studies on repair and reconstruction of wrist ligament injuries were of low level of evidence (LoE IV-V). Analysis of the study types revealed that expert opinions, level of evidence V (55.9%, *n* = 203) was the most frequent study type and no studies of LoE I-II were found between 1985 and April 2017.

In the present study, in general, the Impact factor was relatively low, slightly increasing over years. The Eigenfactor followed the Impact factor. Eigenfactor (Bergstrom et al., 2008) score is correlated with the impact factor and the total citation count for medical journals, but these metrics provide significantly different information. For a given number of citations, citations from more significant journals will result in a higher Eigenfactor score. The result in the present study indicates that citations were given in similar journal that publications about wrist ligament surgery are published in.

### Distribution

Frequency tables for geographic distribution revealed that the United States was the country with the highest number of publications, especially in terms of the two most common journals (*J Hand Surg Am* – 65% and *Hand Clin* – 87%). Frequently recurrent authors were common. Studies with LoE V were particularly common in *Hand Clin* (89%) and *Tech Hand Up Extrem Surg* (75%).

*J Hand Surg Br/Eur, J Wrist Surg* and to some extent also *Arthroscopy* displayed a more diversified geographic frequency distribution and a relatively lower rate of LoE V studies.

To be able to recommend surgery and what kind of procedure in specific cases, the hand surgery community needs larger studies with a randomized prospective assessment. A more diversified geographic distribution in some journals could also be desirable and worthwhile.

Up to this date, the patients´ needs and the surgeon’s specific skills seem to have had a vast impact in choosing type of surgical treatment for SLL, LTL and TFCC/DRUJ injuries (Garcia-Elias et al., 2006; Kitay & Wolfe, 2012; van de Grift & Ritt, 2016; Anderson et al., 2008; Zimmerman & Jupiter, 2014). A surgical method that works in the hands of one surgeon may not work in the hands of others. Therefore studies at a high level of evidence are needed to evaluate different therapeutic methods in terms of wrist ligament injuries. Every clinician has to continuously critically review and re-evaluate his decision of surgical options, especially as we conclude that the level of evidence - in terms of our common recommendations - are really low.

Larger prospective randomized controlled trials (RCTs) are needed to evaluate the outcome of wrist ligament surgery, in terms of minimizing bias and confounding factors. Multi-center studies or non-inferiority studies could also be an option, saving time and costs in the study setting, compared to RCTs.

The literature in terms of wrist ligament repair and reconstruction area has increased dramatically over the last 15 years. Almost 80% of the included studies were published between 2000 and 2016. This systematic review showed that there was no clear trend toward higher level of evidence over time.

As 56.5% of the studies were published in six of the 74 indexed registered orthopaedic journals - and 40% in three of those journals - this could indicate a risk of publication bias.

### Limitations

The search was limited to papers written in English indexed in PubMed, Embase, and Cochrane library. Studies in other languages and cited in other databases were therefore not included in this review. The data extraction was not performed in a blinded fashion, that is, by blacking out authors, title, and so on.

To our knowledge, this is the first systematic review on categorizing the study type and level of evidence of studies on repair and reconstruction of SLL, LTL and TFCC/DRUJ injuries by applying the level of evidence rating system proposed by the Oxford Centre for Evidence-Based Medicine with this extensive electronic search. This extensive search, of course, creates a large number of abstracts to analyze, which creates the possibility that some studies were classified into the wrong category. However, our results are so clear-cut, that wrong categorization in some few cases, with the highest probability, would not affect our conclusion.

## Conclusion

The absolute majority of the therapeutic studies on repair and reconstruction of wrist ligament injuries were of low level of evidence (LoE IV-V; 97%, *n* = 351 (of 362)).

No studies of LoE I-II were found between 1985 and April 2017. There was no clear trend toward higher level of evidence over time. Prospective randomized controlled trials, multi-center studies or non-inferiority studies are needed to better evaluate the outcome of wrist ligament surgery. There is insufficient evidence to recommend one technique over the other in terms of wrist ligament surgery in clinical practice. There is an immense lack of comparison studies with high level of evidence in the area of wrist ligament repair and reconstruction.

## Additional file

Additional file 1: Search strategies. (DOCX 19 kb)
