# Supplementary material for: Level of evidence in wrist ligament repair and reconstruction research: a systematic review
Source: J Exp Orthop. 2018 Jun 7;5:15. doi: 10.1186/s40634-018-0135-7 (PMC5992116; doi:10.1186/s40634-018-0135-7)
Supplement: Supplementary file 1 — Search strategies. (DOCX 19 kb) [file 40634_2018_135_MOESM1_ESM.docx]

**Additional file 1** – Search strategies

**Database:** PubMed

**Date:** 2016-05-24

**No of results:** 1712 ref.
**Search updated:** 2017-04-28, 108 results

| **Search** | **Query** | **Items found** |
| --- | --- | --- |
| [**#36**](http://www.ncbi.nlm.nih.gov/pubmed/advanced) | **Search #27 NOT #31 Filters: Publication date from 1985/01/01; Swedish; English; Danish; Norwegian** | [**1712**](http://www.ncbi.nlm.nih.gov/pubmed/?cmd=HistorySearch&querykey=36) |
| [#32](http://www.ncbi.nlm.nih.gov/pubmed/advanced) | Search #27 NOT #31 | [2167](http://www.ncbi.nlm.nih.gov/pubmed/?cmd=HistorySearch&querykey=32) |
| [#31](http://www.ncbi.nlm.nih.gov/pubmed/advanced) | Search #28 OR #29 OR #30 | [4264368](http://www.ncbi.nlm.nih.gov/pubmed/?cmd=HistorySearch&querykey=31) |
| [#30](http://www.ncbi.nlm.nih.gov/pubmed/advanced) | Search animal[ti] OR animals[ti] OR cadaver*[ti] | [112037](http://www.ncbi.nlm.nih.gov/pubmed/?cmd=HistorySearch&querykey=30) |
| [#29](http://www.ncbi.nlm.nih.gov/pubmed/advanced) | Search ((cadaver[mh]) NOT (cadaver[mh] AND humans[mh])) | [4489](http://www.ncbi.nlm.nih.gov/pubmed/?cmd=HistorySearch&querykey=29) |
| [#28](http://www.ncbi.nlm.nih.gov/pubmed/advanced) | Search ((animals[mh]) NOT (animals[mh] AND humans[mh])) | [4214002](http://www.ncbi.nlm.nih.gov/pubmed/?cmd=HistorySearch&querykey=28) |
| [#27](http://www.ncbi.nlm.nih.gov/pubmed/advanced) | Search #23 AND #26 | [2234](http://www.ncbi.nlm.nih.gov/pubmed/?cmd=HistorySearch&querykey=27) |
| [#26](http://www.ncbi.nlm.nih.gov/pubmed/advanced) | Search #24 OR #25 | [3879928](http://www.ncbi.nlm.nih.gov/pubmed/?cmd=HistorySearch&querykey=26) |
| [#25](http://www.ncbi.nlm.nih.gov/pubmed/advanced) | Search "Surgical Procedures, Operative"[Mesh] | [2639684](http://www.ncbi.nlm.nih.gov/pubmed/?cmd=HistorySearch&querykey=25) |
| [#24](http://www.ncbi.nlm.nih.gov/pubmed/advanced) | Search reconstructive[tiab] OR reconstruction[tiab] OR repair[tiab] OR suture[tiab] OR sutures[tiab] OR reinsertion[tiab] OR surgery[tiab] OR surgical[tiab] OR surgery[sh] | [2679106](http://www.ncbi.nlm.nih.gov/pubmed/?cmd=HistorySearch&querykey=24) |
| [#23](http://www.ncbi.nlm.nih.gov/pubmed/advanced) | Search ((scapholunate OR scapho-lunate OR lunotriquetral OR luno-triquetral) AND ligament) OR triangular fibrocartilage complex OR triangular fibrocartilage ligament OR triangular fibro-cartilage complex OR triangular fibro-cartilage ligament OR distal radioulnar joint OR (wrist[tiab] AND ligament[tiab]) OR (wrist[tiab] AND ligaments[tiab]) | [3350](http://www.ncbi.nlm.nih.gov/pubmed/?cmd=HistorySearch&querykey=23) |

**Database:** Embase (OvidSP) 1974 to 2016 May 23

**Date:** 2016-05-24

**No of results:** 716 ref.
Search updated: 2017-04-28, 68 results

| **#** | **Searches** | **Results** |
| --- | --- | --- |
| 1 | (reconstructive or reconstruction or repair or suture or sutures or reinsertion or surgery or surgical).ti,ab. | 2173297 |
| 2 | exp ligament surgery/ | 5351 |
| 3 | 1 or 2 | 2174237 |
| 4 | ((scapholunate or scapho-lunate or lunotriquetral or luno-triquetral) and ligament).ti,ab. | 728 |
| 5 | ((triangular adj3 fibro?cartilage adj3 complex) or (triangular adj3 fibro?cartilage adj3 ligament)).ti,ab. | 650 |
| 6 | (distal adj3 radio?ulnar adj3 joint).ti,ab. | 1048 |
| 7 | (wrist adj5 ligament?).ti,ab. | 377 |
| 8 | 4 or 5 or 6 or 7 | 2335 |
| 9 | 3 and 8 | 1114 |
| 10 | (animal or animals or cadaver$).ti. | 124081 |
| 11 | (animal not (animal and human)).sh. | 1325043 |
| 12 | 10 or 11 | 1417560 |
| 13 | 9 not 12 | 1085 |
| 14 | limit 13 to (embase and yr="1985 -Current" and (article or conference paper or note or "review")) | 835 |
| **15** | **limit 14 to (danish or english or norwegian or swedish)** | **716** |

**Database:** The Cochrane Library

**Date:** 2016-05-24

**No of results:** 60 ref.

*Cochrane review: 29*

*Other reviews: 3*

*Trial: 28*

*Technology assessments: -*

*Economic evaluations: -*

| **ID** | **Search** | **Hits** |
| --- | --- | --- |
| #1 | reconstructive or reconstruction or repair or suture or sutures or reinsertion or surgery or surgical | 161916 |
| #2 | MeSH descriptor: [Surgical Procedures, Operative] explode all trees | 110760 |
| #3 | #1 or #2 | 200299 |
| #5 | ((scapholunate or scapho-lunate or lunotriquetral or luno-triquetral) and ligament) or triangular fibrocartilage complex or triangular fibrocartilage ligament or triangular fibro-cartilage complex or triangular fibro-cartilage ligament or distal radioulnar joint or (wrist and ligament?) | 70 |
| **#6** | **#3 and #5** | **60** |

**Search updated:** 2017-04-28, 6 results
